# MicroRNA-221/222 Mediates ADSC-Exosome-Induced Cardioprotection Against Ischemia/Reperfusion by Targeting PUMA and ETS-1

**DOI:** 10.3389/fcell.2020.569150

**Published:** 2020-12-03

**Authors:** Tsai-Chun Lai, Tzu-Lin Lee, Yu-Chun Chang, Yu-Chen Chen, Shu-Rung Lin, Shu-Wha Lin, Chi-Ming Pu, Jaw-Shiun Tsai, Yuh-Lien Chen

**Affiliations:** ^1^Department of Anatomy and Cell Biology, College of Medicine, National Taiwan University, Taipei, Taiwan; ^2^Department of Bioscience Technology, College of Science, Chung Yuan Christian University, Taoyuan, Taiwan; ^3^Center for Nanotechnology and Center for Biomedical Technology, Chung Yuan Christian University, Taoyuan, Taiwan; ^4^Department of Clinical Laboratory Sciences and Medical Biotechnology, College of Medicine, National Taiwan University, Taipei, Taiwan; ^5^Department of Anatomy and Cell Biology, College of Medicine, National Taiwan University, Taipei, Taiwan; ^6^Division of Plastic Surgery, Department of Surgery, Cathay General Hospital, Taipei, Taiwan; ^7^Department of Family Medicine, National Taiwan University Hospital, Taipei, Taiwan; ^8^Center for Complementary and Integrated Medicine, National Taiwan University Hospital, Taipei, Taiwan

**Keywords:** ischemia – reperfusion, ADSC, adipose derived mesenchymal stem cell, exosome, miR-221 and 222, apoptosis, hypertrophy

## Abstract

Cardiovascular disease is a major health problem in industrialized and developing countries and is the leading cause of death and disability. Myocardial ischemia/reperfusion (I/R) causes cardiomyocyte damage such as apoptosis and hypertrophy. The purpose of this study was to investigate the effects of exosomes from adipose-derived stem cells (ADSC-Exo) on hearts from I/R mice and to explore the underlying mechanisms. ADSC-Exo significantly decreased I/R-induced cardiomyocyte apoptosis and hypertrophy, as detected by TdT-mediated dUTP nick end-labeling (TUNEL) and wheat germ agglutinin (WGA) staining, respectively. In addition, the expression of apoptosis-related proteins p-p53 and PUMA and hypertrophy-related proteins ETS-1 and ANP were significantly reduced in the cardiomyocytes of ADSC-Exo-treated I/R mice compared to those of control mice. Both PUMA and ETS-1 are reported to be target genes for miR-221/222. I/R operation significantly reduced miR-221/222 expression, while ADSC-Exo treatment increased miR-221/222 expression, as detected by RT-qPCR. We also observed that cardiac I/R operation markedly increased cell apoptosis and hypertrophy in miR-221/222 knockout (KO) mice, while ADSC-Exo reduced the effects of I/R operation. Furthermore, ADSC-Exo protected H9c2 cardiomyocytes from H_2_O_2_-induced damage by reducing apoptosis and hypertrophy *in vitro*. H_2_O_2_ treatment significantly reduced miR-221/222 expression, while ADSC-Exo treatment reversed this effect in H9c2 cells. ADSC-Exo treatment decreased H_2_O_2_-induced PUMA and ETS-1 expression. Compared with control treatment, I/R treatment significantly reduced p-AKT and increased p-p65, while ADSC-Exo and miR-221/222 mimics attenuated these effects. The AKT activator SC79 and p65 inhibitor Bay 11-7082 reduced H_2_O_2_-induced cell apoptosis and hypertrophy. Based on these findings, ADSC-Exo prevents cardiac I/R injury through the miR-221/miR-222/PUMA/ETS-1 pathway. Therefore, ADSC-Exo is an effective inhibitor of I/R-induced heart injury.

## Introduction

For patients with myocardial infarction (MI), thrombolysis or percutaneous coronary intervention (PCI) is a timely and effective myocardial reperfusion treatment that can reduce acute myocardial ischemic injury and limit the size of the MI (Hausenloy and Yellon, 2013). Although a safe cardiac surgery technique can improve surgical outcomes, the reperfusion process itself can induce cardiac cell death, known as cardiac ischemia/reperfusion (I/R) injury (Dhalla et al., 2000; Hausenloy and Yellon, 2013). I/R injury includes a series of complex events, including increased oxidative stress, induced inflammation, triggered myocardial apoptosis and hypertrophy, and can exacerbate heart damage and account for up to 50% of the infarct size (Yellon and Hausenloy, 2007; Heusch et al., 2013; Santos-Gallego et al., 2016). Although efforts have been made to understand the mechanisms that cause cardiac I/R injury, the molecular and cellular events that regulate cardiac injury after I/R are still only partially understood. Therefore, new pharmacological interventions are needed to prevent I/R injury and potentially improve the clinical outcomes of patients with acute MI undergoing PCI (Heusch and Gersh, 2017; Li Y. et al., 2019).

Adipose-derived stem cells (ADSCs) play a vital role in wound repair. Recent studies have shown that exosomes, membrane lipid nanovesicles with a diameter of 30–100 nm, secreted by stem cells contribute to paracrine signaling (Hu et al., 2016; Eguchi et al., 2019). The exosomes of these cells have the advantages of high stability, non-immune rejection, homing, and easy control of dose and concentration (Hu et al., 2016). However, there is no direct evidence to prove their mechanism of action. According to previous studies, exosomes are important mediators of intercellular signal transduction, as there are multiple microRNAs (miRNAs) and proteins in exosomes (Waldenström and Ronquist, 2014). miRNA are small (∼22 nt) non-coding RNAs that negatively regulate the expression of protein-coding genes by inhibiting mRNA translation (Valencia-Sanchez et al., 2006). MiRNA expression is associated with cardiac events such as electrical signaling, muscle contraction, heart growth, and morphogenesis (Yang et al., 2007; Zhao et al., 2007). Increasing evidence indicates that changes in miRNA expression profiles can often be detected in heart diseases such as MI and heart failure (Abdellatif, 2012). Therefore, it may be possible to manipulate miRNAs to achieve therapeutic effects (Barraclough et al., 2019). miR-221 and miR-222 (miR-221/222 clusters) share the same ‘seed’ sequence, evolutionarily conserved and short regions at their 5′ ends through which they bind their target sites in the mRNA 3′ untranslated region (UTR) (Song et al., 2017). This cluster is dysregulated in human cancers, atherosclerotic vascular remodeling, and viral myocarditis (Chistiakov et al., 2015; Corsten et al., 2015). MiR-221/222 targets p53 upregulated modulator of apoptosis (PUMA) and acts as an apoptosis regulator for epithelial cells (Zhang et al., 2010). miR-221/222 also targets ETS protooncogene 1 (ETS-1) (Zhu et al., 2011). However, the role of miR-221/miR-222 in the heart during I/R is still unknown, and further research is still in progress. Previous studies have shown that PUMA and ETS-1 can regulate apoptosis and hypertrophy, respectively (Zhan et al., 2005; Mandl et al., 2011). Taking all these factors into consideration, the purpose of this study was to examine whether miR-221/222 can participate in the regulation of H9c2 myocardial cell apoptosis and hypertrophy and MI by targeting PUMA and ETS-1. Our results indicate that PUMA and ETS-1 participate in H_2_O_2_-induced apoptosis and hypertrophy by reducing miR-221/222 expression in H9c2 cells. miR-221/222 overexpression antagonizes these phenomena in cell models and mouse I/R models. We also found that exosomes derived from ADSCs can reduce cell apoptosis and cell hypertrophy, which is beneficial for I/R injury. These data provide strong *in vitro* and *in vitro* evidence that ADSC-Exo has clinical application prospects in I/R healing.

## Materials and Methods

### *In vivo* Myocardial I/R Model and ADSC-Exo Transfer

Male C57BL/6 wild-type (WT) mice and miR-221/222 knockout (KO) mice (body weight: 25–30 gm; age: 8–12 weeks) were used in this study. We generated miR-221/222 KO mice by deleting the X-linked miR-221/222 gene and bred them for 10 generations on a C57BL/6 background. Mice were anesthetized with isoflurane, and the heart was subjected to I/R surgery. In short, ischemia was achieved by ligating the anterior descending branch of the left anterior descending coronary artery (LAD) with a 7-0 nylon suture and placing a silicone tube (OD 86 mm) 1 mm below it. The efficacy of the occlusion was verified by blanching the ventricle at the distal end of the ligation. Then, 25 min after occlusion, ADSC-Exo (100 μg protein in 50 μL) were evenly intramuscularly injected into the border zone of the anterior wall of the left ventricle at five positions. After 30 min of occlusion, the silicon tubing was removed for reperfusion. After 30 min of ischemia and 3 h of reperfusion, all mice were reanesthetized, and the chest was reopened. Heart and blood samples were obtained for further analysis. In the sham group, the heart was exposed without ligating the LAD. All animal experiments were conducted in accordance with the guidelines for animal care of the National Taiwan University (IACUC Approval No: 20150502) and complied with the Guide for the Care and Use of Laboratory Animals, NIH publication No. 86–23, revised in 1985.

### Physiological Assessment of Cardiac Function

The influence of I/R and ADSC-Exo on cardiac function was evaluated by echocardiography. Echocardiography was performed with a dedicated small-animal high-resolution ultrasound system (Prospect, S-Sharp, Taipei, Taiwan), equipped with a 40-MHz single-element transducer. M-mode tracings recorded at the level of the papillary muscle of the left ventricle from the long-axis view was used to evaluate fractional shortening (FS) and ejection fraction (EF).

### Cell Culture

Embryonic rat heart-derived H9c2 cells were purchased from the American Type Culture Collection (VA, United States) and were cultured in Dulbecco’s modified Eagle’s medium (DMEM, GIBCO, NY, United States) supplemented with 10% fetal bovine serum (FBS), 110 mg/mL sodium pyruvate, 100 U/mL penicillin, and 100 μg/mL streptomycin at 37°C in a humidified atmosphere containing 5% CO_2_. Unless otherwise stated, cells were treated with 50 μM H_2_O_2_ for 48 h. H9c2 cells were pretreated with ADSC-Exo (2 μg/mL) for 4 h and then treated with 50 μM H_2_O_2_ for 48 h. Human ADSCs were purchased from LONZA (Basel, Switzerland). ADSCs were cultured in DMEM containing 20% FBS and penicillin/streptomycin. Cells between passages 3 and 8 were used for all experiments.

### Extraction, Purification, and Characterization of ADSC-Exo

ADSCs were trypsinized and seeded at 5 × 10^5^ cells in a 10-cm dish. After 24 h, the culture medium was collected and centrifuged at 3,000 *g* for 15 min to remove cells and cell debris. ADSC-Exo were isolated from 10 mL culture media using 2 mL ExoQuick-TC^TM^ Exosome Precipitation Solution per the manufacturer’s instructions (System Biosciences, CA, United States). After overnight incubation at 4°C, the mixture was centrifuged at 10,000 *g* for 30 min at 4°C. The pellet was washed with 1 mL PBS and centrifuged at 4°C for 15 min at 10,000 *g*. Then, the purified ADSC-Exos were completely suspended in 50 μL PBS and stored at −80°C for further study. The protein content of the exosomes was quantified using the Bradford method. The purified ADSC-Exo was characterized using the biomarker CD63 and CD9 by Western blot. The morphology and size of ADSC-Exo were observed under a transmission electron microscope (Hitachi, Tokyo, Japan). Nanoparticle tracking analysis (NTA) was used to evaluate the concentration and size distribution of ADSC-Exo. The detailed methods and results for characterization of ADSC-Exo were described in the Supplementary Data. Cardiac and cellular uptake of ADSC-Exo were examined using the PKH-26 labeling kit (Sigma, MO, United States) and observed under a confocal microscope.

### Detection of Lactate Dehydrogenase (LDH)

The myocardial damage was assessed by measuring the level of LDH in the blood. A commercial LDH assay kit (Abcam, MA, United States) was used. All procedures were performed in accordance with the manufacturer’s instructions.

### Measurement of Reactive Oxygen Species (ROS) Production

2′,7′-Dichlorodihydrofluorescein diacetate (DCFDA) (Thermo, MA, United States) and dihydroethidium (DHE) (Invitrogen, MA, United States) were used to detect the levels of intracellular oxidative free radicals and superoxide anions, respectively. Cryostat sections of the heart were incubated with 2 μM DHE in the dark at 37°C for 20 min. H9c2 cells were stained with DCFDA (10 μM) in a dark environment at 37°C for 1 h. Oxidative stress was examined and observed on a fluorescence microscope. DHE fluorescent intensity was further quantified by ImageJ (NIH, MD, United States), and the relative expression was expressed as fold change relative to the control.

### Assessment of Apoptosis by TdT-Mediated dUTP Nick End-Labeling (TUNEL) Assay

Five-micrometer paraffin sections of heart samples obtained from I/R or cultured cardiomyocytes were used to study apoptosis. According to the manufacturer’s instructions, apoptosis was detected by a TUNEL assay kit (*In Situ* Cell Death Detection Kit, Roche, CA, United States). The percentage of TUNEL-positive nuclei relative to the total nuclei was determined blindly by calculating five randomly selected 40x fields of view on each cover glass on each slide. In addition, TUNEL staining was used to evaluate H9c2 cell apoptosis by flow cytometry.

### Western Blot Analysis

Western blot analysis was performed as described previously (Liang et al., 2011). The heart was crushed in liquid nitrogen and homogenized in RIPA buffer (TOOLS, New Taipei City, Taiwan) supplemented with protease inhibitor cocktail (Genestar Biotechnology, Taiwan). In addition, to prepare cell lysates, cells were lysed in RIPA buffer with protease inhibitor cocktail at 4°C for 1 h, followed by centrifugation at 14000 *g* for 15 min at 4°C, and the supernatant was retained. An equal amount of the supernatant (20 μg protein) was subjected to 10% SDS-PAGE and then transferred to a PVDF membrane (Merck, NJ, United States). The membrane was then incubated with 5% skim milk in Tris buffered saline containing 0.2% Tween 20 (TBST) for 1 h at room temperature (RT). The blots were incubated with primary antibodies as follows: ETS-1, PUMA, and CD9 were purchased from Abcam, CD73 and CD63 were purchased from GeneTex (Hsinchu, Taiwan), HIF-1α and phosphorylated p53 were purchased from Cell Signaling (MA, United States), BCL-2 was purchased from BD (CA, United States), and ANP was purchased from Santa Cruz (TX, United States) overnight at 4°C. Then, the cells were incubated with horseradish peroxidase-conjugated goat anti-rabbit IgG antibody for 1 h (Jackson ImmunoResearch, CA, United States). Chemiluminescence Reagent Plus (NEN, MA, United States) was used to develop the signal, and images were captured by a Biospectrum 600 imaging system (UVP, CA, United States). Quantification of the intensity of each band was performed with ImageJ. Anti-GAPDH antibody (Proteintech, IL, United States) was used as a loading control.

### Wheat Germ Agglutinin (WGA) Staining

The heart was cut in half by forming a transverse slice between the atrioventricular sulcus and the apex. The base specimen was fixed in 10% formalin buffer, embedded in paraffin, and cut into 5-μm thick sections. The cross-sectional area of cardiomyocytes was measured in images captured in sections stained with 5 μM WGA (Thermo, MA, United States). H9c2 cells were exposed to 50 μM H_2_O_2_ or the indicated treatment for 48 h. After fixation with 4% paraformaldehyde, cells were incubated with 5 μM WGA for 10 min at RT. Images were observed by a fluorescence microscope. The surface area was measured with ImageJ to check for hypertrophy, and the value was expressed as fold change relative to the control.

### Cell Volume Measurement

H9c2 cells were treated with 50 μM H_2_O_2_ for 48 h or the indicated treatment and then trypsinized and suspended in PBS for flow cytometry analysis. The cell volume was analyzed by forward scatter (FSC) mode by flow cytometry, and the value was expressed as the volume change (%) compared to the that in untreated group. In addition, cells undergoing the indicated treatments were photographed by an inverted microscope. Images were subjected to measurement of the surface area by ImageJ, and the value was expressed as fold change relative to the control.

### Immunostaining

Cultured cells or heart paraffin sections were incubated with specific primary antibodies (dilution 1:100 for anti-PUMA antibody, 1:100 for anti-ETS-1 antibody) at 4°C overnight. Sections were then incubated with biotin-conjugated goat anti-rabbit IgG (1:200 dilution; Vector Lab, United Kingdom) for 1 h at RT. Finally, the reaction was developed with DAB, and the cells were counterstained with hematoxylin and examined by light microscopy.

### RNA Isolation and Quantitative Reverse Transcription PCR (RT-qPCR)

Total RNA was isolated from cells using TRIzol reagent (Thermo) according to the manufacturer’s instructions. The TaqMan MicroRNA Reverse Transcription Kit (Invitrogen, CA, United States) was used to obtain the cDNA template. To analyze miR-221/222 expression, RT-qPCR was performed by using TaqMan Universal PCR Master Mix (Applied Biosystems, CA, United States). TaqMan microRNA assay kits for miR-221 (000524), miR-222 (002276), and RNU6B (001973) were obtained from Applied Biosystems. All reactions were performed in triplicate. The amount of miR-221/222 was determined by standardizing to that of RNU6B.

### Transient Transfection

H9c2 cells were seeded in a 6-well plate at a concentration of 3 × 10^5^ cells per well and transfected with specific miR-221/222 mimics (100 nM) (Dharmacon, CO, United States) or FAM-labeled mimics (GenePharma, Shanghai, China) or a duplex RNA inhibitor (Dharmacon) using Lipofectamine 3000 reagent according to the manufacturer’s protocol followed by the analysis of ETS-1 and PUMA expression, apoptosis, and hypertrophy. Non-targeting sequences were used as negative controls.

PUMA or ETS-1 knockdown was performed with specific siRNA (1 μM) using Lipofectamine 3000. The downregulation of ETS-1 and PUMA was confirmed by Western blot analysis. In addition, full-length cDNA coding rat ETS-1 and PUMA were purchased from Dharmacon. Transient transfection of H9c2 cells was performed with Lipofectamine 3000 reagent.

*In vivo* transfection was performed by TurboFect *in vivo* transfection reagent (Thermo), and miR-221/222 mimics or inhibitors (50 pmole) were prepared at a concentration of 50 μL according to the manufacturer’s instructions. The *in vivo* transfection process followed the protocol of I/R with ADSC-Exo injection. The 50 μL mixture of mimics or inhibitors was injected uniformly intramuscularly into the border zone of the anterior wall of the left ventricle at five positions.

### Luciferase Reporter Assay

The WT and mutant (MUT) PUMA 3′ UTR luciferase reporter gene plasmids were generated by Promega (WI, United States). The cells were then cotransfected with miR-NC or miR-221/222 mimics with the WT or MUT PUMA 3’ UTR reporter plasmid using Lipofectamine 3000 reagent. After 48 h incubation at 37°C, a dual-luciferase assay system (Promega) was used to measure the relative luciferase activity. In addition, WT and MUT of ETS-1 3’ UTR luciferase reporter gene plasmids were generated by Promega. Similar procedures were performed as described above.

### Annexin V/Propidium Iodide (PI) Dual Staining

H9c2 cells were seeded in 6-well plates at a density of 3 × 10^5^ cells per well. After performing various treatments on different groups, the percentage of apoptotic cells was detected using the Annexin V-FITC/PI Apoptosis Detection Kit (BD) according to the manufacturer’s protocol and examined by a FACSCanto II flow cytometer (BD). Annexin V-positive and PI-negative populations represent early apoptotic cells, while annexin V-positive and PI-positive populations represent late apoptotic cells. According to the previous report, these populations are usually selected as apoptosis cells (Xiao et al., 2016).

### Statistics

Quantitative data were obtained from the indicated number of experiments and expressed as the means ± SD. Differences between 2 groups were calculated using the Student’s *t* test. The statistical significance of differences between multiple experimental groups was determined by one-way analysis of variance (ANOVA) and followed by Dunnett *post hoc* test. SPSS (version 17.0; IBM, NY, United States) was used for data analysis. A value of *P* < 0.05 was considered statistically significant.

## Results

### The Cardioprotective Effects of ADSC-Exo Were Associated With Reduced Apoptosis and Hypertrophy in I/R-Induced Heart Injury

**FIGURE 1:**
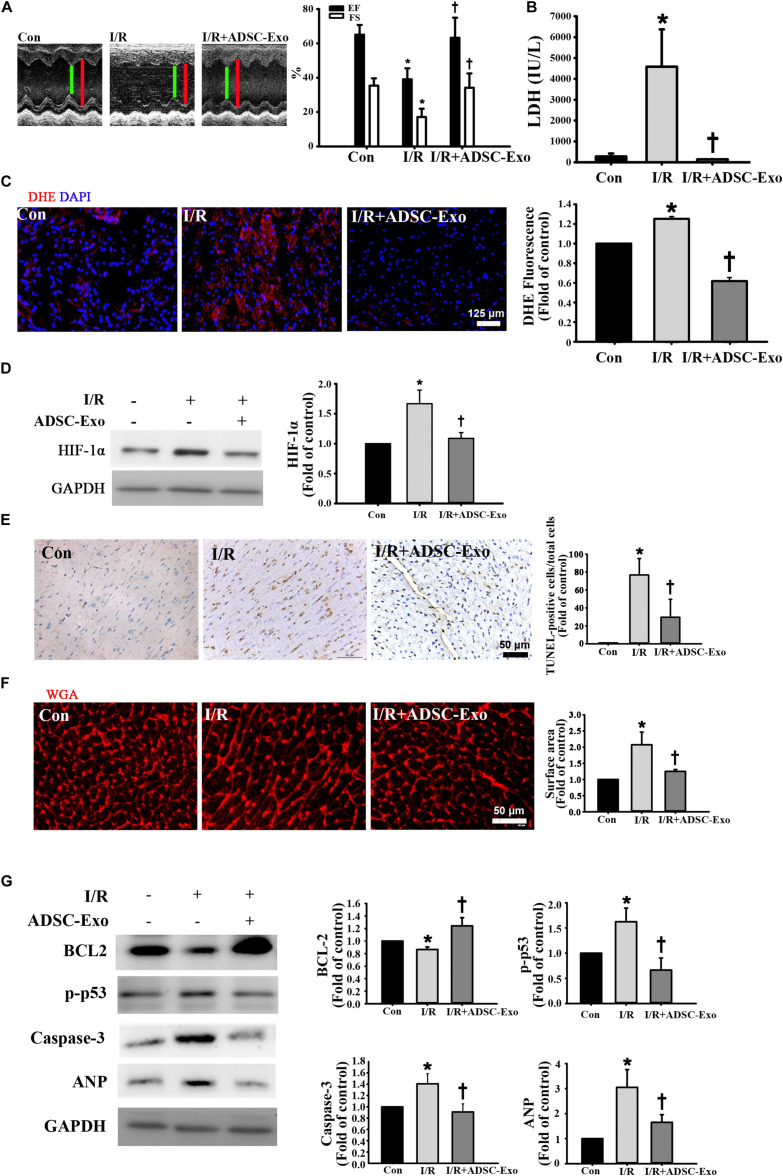
ADSC-Exo treatment attenuated I/R-induced myocardial apoptosis and hypertrophy *in vivo*. Mice were subjected to ischemia for 30 min followed by reperfusion for 3 h. In the I/R + ADSC-Exo mouse model, 25 min after occlusion, ADSC-Exo (100 μg protein in 50 μL) were injected uniformly intramuscularly into five positions in the border zone of the left ventricular anterior wall and then perfused for 3 h. **(A)** Representative M-mode images from control mice, I/R-treated mice, and ADSC-Exo-treated I/R mice. The left ventricular ejection fraction (EF) and fractional shortening (FS) in the ADSC-Exo-treated I/R mice were significantly increased compared with those in I/R-treated mice. **(B)** The level of lactate dehydrogenase (LDH) release. **(C)** Measurement of intracellular ROS by DHE staining. Scale bar, 125 μm. A quantitative analysis of DHE fluorescence is shown. **(D)** Western blot analysis of the level of HIF-1α expression. **(E)** Effect of ADSC-Exo on apoptosis after myocardial I/R injury by TUNEL assay. **(F)** Quantitative analysis of cardiomyocyte cross-sectional area by WGA staining is shown. Heart sections were stained with WGA staining, and the cardiomyocyte cross-sectional area was determined. Cell image analysis of the relative cell surface area (cell size) was performed. Scale bar, 50 μm. **(G)** The levels of BCL2, p-p53, caspase-3, and ANP protein were evaluated by Western blot analysis. Data are expressed as the mean ± SD (*n* = 3). *P <0.05 vs. the control group; ^†^*P* <0.05 vs. I/R group.

In the first set of experiments, we investigated whether ADSC-Exo improved cardiac function in I/R mice. Figure 1A shows representative M-mode images from control mice, I/R-treated mice, and ADSC-Exo-treated I/R mice. The level of EF and the FS in I/R animals were markedly decreased compared with those in the control animals. By contrast, EF and FS in the ADSC-Exo-treated mice were significantly increased compared with those in I/R-treated mice. LDH release is an indicator of cell damage. As shown in Figure 1B, after 30 min of myocardial ischemia followed by reperfusion for 3 h, the release of LDH in the plasma of the I/R group increased significantly. ADSC-Exo significantly reduced the release of LDH. Oxidative stress plays a crucial role in I/R-induced cardiac injury. Therefore, we tried to study the effect of ADSC-Exo on ROS production. As shown in Figure 1C, DHE-positive cells were clearly present in the I/R group. ADSC-Exo greatly reduced ROS production. Similarly, Western blot was used to confirm that I/R increased HIF-1α expression and ADSC-Exo decreased HIF-1 expression (Figure 1D).

Next, we examined the presence or absence of ADSC-Exo-treated I/R-induced cardiomyocyte apoptosis and hypertrophy. I/R induced significant apoptosis, while ADSC-Exo reduced this effect, as detected by TUNEL analysis (Figure 1E). In addition, WGA staining showed that I/R induced cell hypertrophy, while ADSC-Exo reduced hypertrophy compared to that in the I/R group (Figure 1F). In addition to the direct detection of cardiomyocyte apoptosis and hypertrophy by TUNEL and WGA staining, further molecular mechanism studies include the detection of apoptosis markers (such as p-p53, caspase-3, and BCL2) and hypertrophy markers (such as ANP). As shown in Figure 1G, I/R treatment significantly reduced BCL2 levels and increased the expression of p-p53, caspase-3, and ANP, while ADSC-Exo treatment significantly reversed this effect.

### ADSC-Exo Protected Against Myocardial I/R Injury in Mice by miR-221/222

**FIGURE 2:**
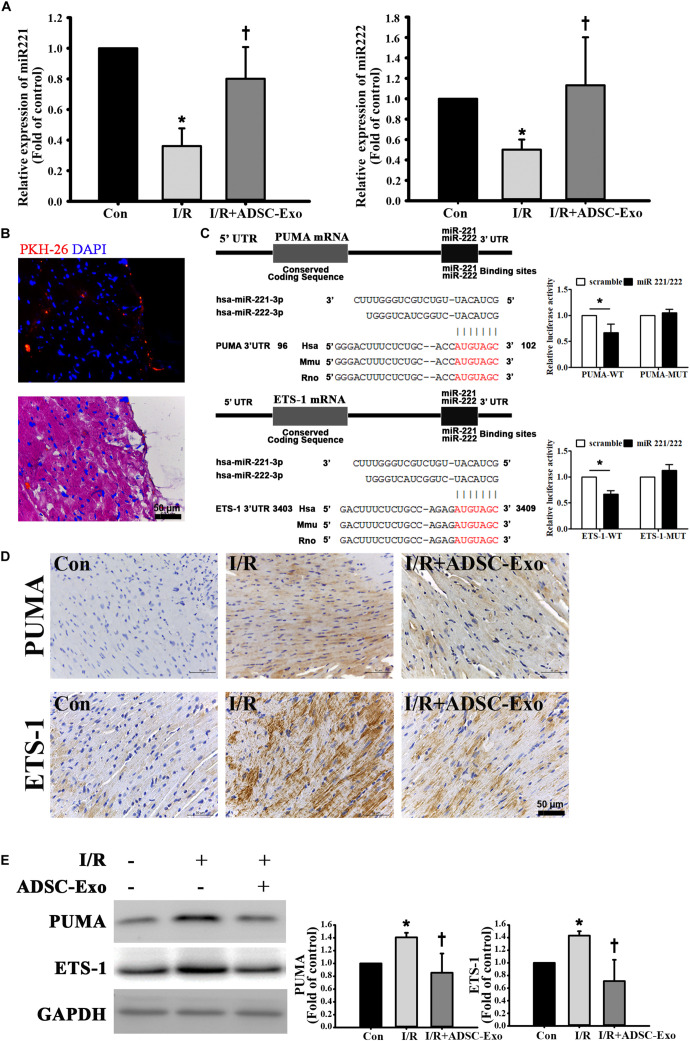
ADSC-Exo protected against myocardial I/R injury in mice by miR-221/222. Mice were subjected to ischemia for 30 min followed by reperfusion for 3 h. In the I/R+ADSC-Exo mouse model, 25 min after occlusion, ADSC-Exo (100 μg protein in 50 μL) were evenly intramuscularly injected into the border zone of the anterior wall of the left ventricle at five sites, followed by reperfusion for 3 h. **(A)** The levels of miR-221/222 were determined by RT-qPCR. **(B)** PKH-26-labeled ADSC-Exo (red) were present in cardiac tissues. **(C)** Potential binding sites of miR-221/222 in the 3′ UTRs of PUMA and ETS-1 mRNA. Luciferase activity analysis of cotransfection of wild-type PUMA 3′ UTR (PUMA-WT) or mutant-type PUMA 3′ UTR (PUMA-MUT) with miR-221/222 mimics or negative control (scramble). In addition, luciferase activity analysis of cells cotransfected with wild-type ETS-1 3′ UTR (ETS-1-WT) or mutant-type ETS-1 3′ UTR (ETS-1-MUT) with miR-221/222 mimics or negative control (scramble) was performed. **(D)** Expression of PUMA and ETS-1 in cardiac tissues, as detected by immunohistochemistry. The positive reaction product was indicated by brown color. Nuclei were counterstained with hematoxylin solution. **(E)** Western blot analysis of the levels of PUMA and ETS-1 expression. Data are expressed as the mean ± SD (*n* = 3). GAPDH was used as the control. *P < 0.05 vs. the control group; ^†^*P* < 0.05 vs. the I/R group.

miR-221/222 expression was markedly downregulated in I/R-treated hearts by RT-qPCR. Injecting ADSC-Exo into the myocardium significantly increased miR-221/222 expression (Figure 2A). ADSC-Exo were clearly present in cardiac tissues (Figure 2B). Bioinformatics analysis using the “miRanda” miRNA target prediction program showed that PUMA and ETS-1 are potential target genes for miR-221/222. Specifically, the 3′ UTRs of PUMA mRNA and ETS-1 mRNA contain putative miR-221/222 binding sites. A dual-luciferase reporter assay verified that miR-221/222 impaired the luciferase activity of the wild-type PUMA 3′ UTR and ETS-1 3′ UTR (WT) but not the MUT 3′ UTR of PUMA and ETS-1 in cells (Figure 2C). As shown in Figure 2D, PUMA and ETS-1 staining was not observed by immunohistochemistry in the control group, whereas strong staining was observed on cardiac tissues in the I/R treatment group. In contrast, administration of ADSC-Exo resulted in weaker PUMA and ETS-1 staining in I/R-treated mice. Similarly, the data were further confirmed by Western blot analysis (Figure 2E). The results showed that the protein levels of PUMA and ETS-1 were increased by I/R treatment, while ADSC-Exo reduced the expression in response to I/R.

### ADSC-Exo Reduced Apoptosis and Hypertrophy in H_2_O_2_-Treated H9c2 Cells

**FIGURE 3:**
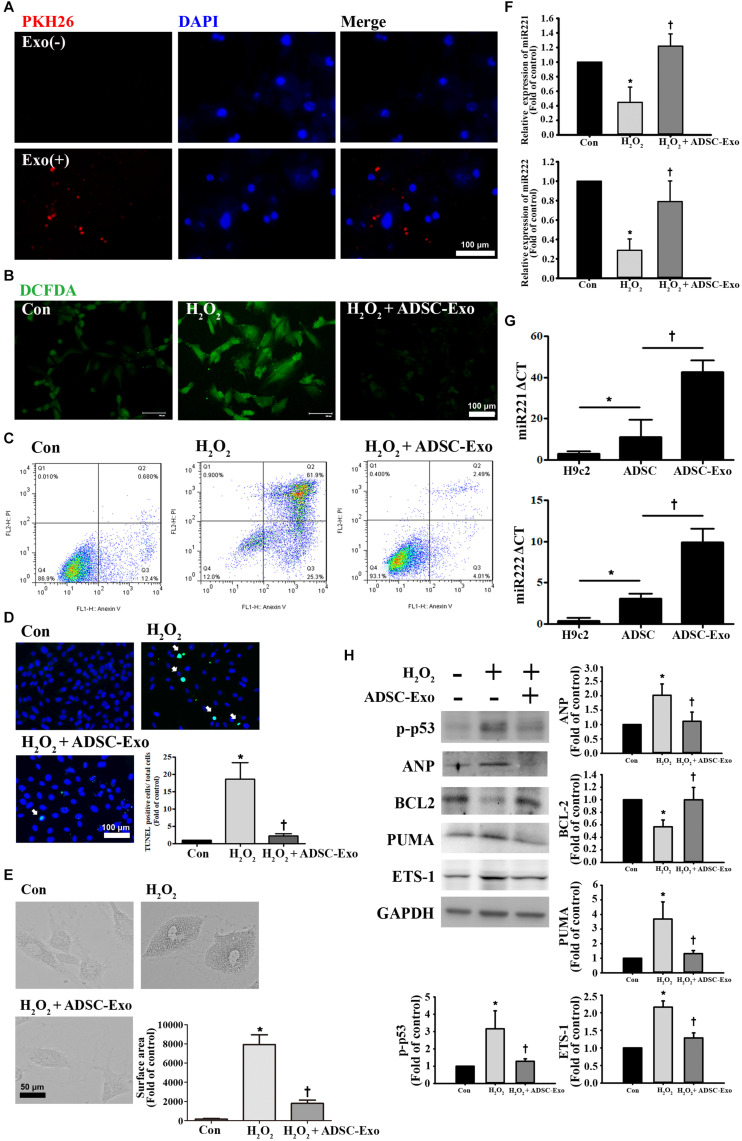
ADSC-Exo attenuated H_2_O_2_-induced apoptosis and hypertrophy by downregulating the expression of PUMA and ETS-1 in H9c2 cells. Cardiomyocytes were pretreated with or without ADSC-Exos (2 μg/mL) for 4 h and then exposed to 50 μM H_2_O_2_ for 48 h. **(A)** PKH26-labeled exosomes (red) were taken up by H9c2 cells. The blue color represents the cell nucleus. Scale bar, 100 μm. **(B)** H_2_O_2_ increased ROS production, while ADSC-Exo significantly reduced ROS production by DCFDA assay. Green color represents ROS-positive cells. Scale bar, 100 μm. **(C)** Analysis of apoptotic cells by flow cytometry using annexin V/PI staining. **(D)** Apoptosis was examined by TUNEL assay. Green, TUNEL-positive nuclei; blue, nuclei. A quantitative analysis of cell death is shown. Treatment with ADSC-Exo reduced H_2_O_2_-induced apoptosis. Scale bar, 100 μm. **(E)** H_2_O_2_ induced cell hypertrophy, and ADSC-Exo treatment reduced cell hypertrophy by observation with an inverted microscope. A quantitative analysis of cell surface area is shown. Scale bar, 50 μm. **(F)** Compared with those in the control group, the miR-221/222 levels in the H_2_O_2_ treatment group were significantly downregulated, as indicated by RT-qPCR, while ADSC-Exo increased the expression. **(G)** The levels of miR-221/222 in ADSCs were significantly higher than those in the H9c2 cells. Importantly, the levels of miR-221/222 in ADSC-Exo were significantly higher than those in the ADSCs. cells. **(H)** The levels of apoptosis markers (p-p53, BCL2, and PUMA) and hypertrophy markers (ETS-1 and ANP) were examined by Western blot analysis. GAPDH was used as the loading control. Data are expressed as the mean ± SD (*n* = 3). *P < 0.05 vs. the control group; ^†^*P* < 0.05 vs. H_2_O_2_.

To further understand the protective mechanism of ADSC-Exo against myocardial I/R injury, H9c2 cardiomyocytes treated with H_2_O_2_ were used to mimic myocardial I/R injury *in vitro*. It is well known that oxidative stress is an important factor in inducing apoptosis and hypertrophy in cardiomyocytes (Tsutsui et al., 2011). H9c2 cells were exposed to different H_2_O_2_ concentrations from 25 to 400 μM, and H_2_O_2_ reduced cell viability in a concentration-dependent manner (data not shown). PKH26-labeled exosomes were taken up by H9c2 cells (Figure 3A). H9c2 cells treated with 50 μM H_2_O_2_ for 24 h increased ROS production, while ADSC-Exo significantly reduced ROS production according to the DCFDA assay (Figure 3B). Moreover, apoptosis levels were detected by annexin V/PI staining by flow cytometry (Figure 3C). H_2_O_2_ caused an increase in apoptosis, whereas ADSC-Exo decreased apoptosis. Apoptosis was further confirmed by the TUNEL assay, and ADSC-Exo treatment reduced apoptosis induced by H_2_O_2_ (Figure 3D). In addition, H_2_O_2_ induced cell hypertrophy, whereas ADSC-Exo treatment reduced cell hypertrophy (Figure 3E). Because ADSC-Exo treatment increased the miR-221/222 expression in I/R-treated hearts, we evaluated whether miR-221/222 protects cardiomyocytes from H_2_O_2_-induced apoptosis and hypertrophy. According to the RT-qPCR results, miR-221/222 levels were significantly downregulated in the H_2_O_2_ treatment group compared with the control group, while ADSC-Exo increased miR-221/222 expression (Figure 3F). In addition, compared with ADSCs, ADSC-Exo contains a large amount of miR-221 and miR-222 (Figure 3G). To further investigate the molecular mechanisms involved, the expression levels of apoptotic proteins and markers of hypertrophy (such as p-p53, BCL2, and ANP) were measured. As shown in Figure 3H, H_2_O_2_ treatment significantly increased the levels of p-p53 and ANP and decreased the levels of BCL2, while the presence of ADSC-Exo significantly attenuated the increase in the levels of p-p53 and ANP. In addition, Western blot analysis indicated that H_2_O_2_ treatment can increase the levels of PUMA and ETS-1 expression. In contrast, ADSC-Exo reduced the expression. Taken together, these data reveal that the administration of ADSC-Exo results in a reduction in apoptosis and hypertrophy induced by H_2_O_2_. These results indicate that miR-221/222 is an effective regulator of cardiomyocyte apoptosis and hypertrophy.

### miR-221/222 Was Involved in ADSC-Exo-Mediated Inhibition of Apoptosis and Hypertrophy in H_2_O_2_-Treated Cardiomyocytes

**FIGURE 4:**
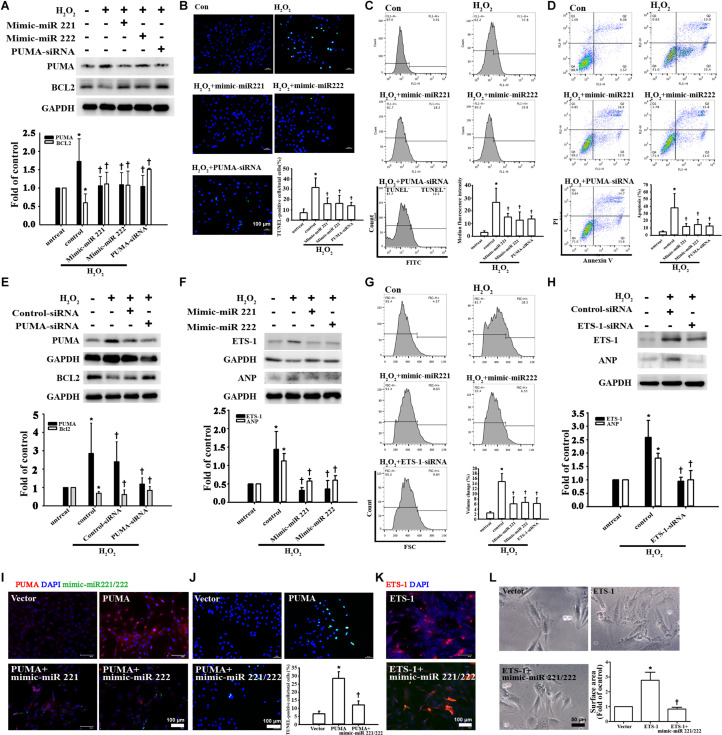
miR-221/222 was involved in ADSC-Exo-mediated inhibition of H_2_O_2_-induced cardiomyocyte apoptosis and hypertrophy. H9c2 cells were transfected with miR-221/222 mimics or siRNA targeting PUMA and ETS-1 for 48 h and then exposed to 50 μM H_2_O_2_ for 48 h. **(A)** Western blot analysis of PUMA and BCL2 protein levels using miR-221/222 mimics or PUMA siRNA. **(B–D)** Transfection with miR-221/222 mimics or PUMA siRNA significantly decreased H_2_O_2_-induced apoptosis, as examined by TUNEL assay using fluorescence microscopy **(B)** or flow cytometry **(C)** and by annexin V/PI staining using flow cytometry **(D)**. **(E)** Knockdown of PUMA in H9c2 cells exposed to H_2_O_2_ increased BCL2 expression and decreased apoptosis. **(F)** The miR-221/222 mimics significantly reduced the expression of ETS-1 and ANP in H_2_O_2_-treated cardiomyocytes. **(G)** Transfection with miR-221/222 mimics also significantly decreased H_2_O_2_-induced hypertrophy by flow cytometry. Forward scatter (FSC) represents cell size. **(H)** Knockdown of ETS-1 in H9c2 cells exposed to H_2_O_2_ reduced ANP expression by Western blot. **(I)** Overexpression of PUMA was confirmed by immunofluorescent staining, and transfection with miR-221/222 mimics decreased PUMA expression. **(J)** PUMA overexpression-induced apoptosis, as examined by TUNEL assay, while transfection with miR-221/222 mimics decreased apoptosis. **(K)** Overexpression of ETS-1 was confirmed by immunofluorescent staining, and transfection with miR-221/222 mimics decreased ETS-1 expression. **(L)** ETS-1 overexpression induced hypertrophy, while transfection with miR-221/222 mimics reversed this effect. A quantitative analysis of cell surface area is shown. Data are expressed as the mean ± SD (*n* = 3). *P < 0.05 vs. the control (untreated) group; ^†^*P* < 0.05 vs. H_2_O_2_.

To demonstrate the effects of miR-221/222 on apoptosis and hypertrophy, the expression levels of the apoptosis marker PUMA and the hypertrophy marker ETS-1 were measured in cardiomyocytes transfected with miR-221/222 mimics. miR-221/222 mimics significantly reduced PUMA expression and increased BCL2 expression in H_2_O_2_-treated cardiomyocytes (Figure 4A). TUNEL assay evaluated by fluorescence microscopy or flow cytometry and annexin V/PI staining evaluated by flow cytometry showed that transfection with miR-221/222 mimics also significantly reduced H_2_O_2_-induced apoptosis compared to that in the control group (Figures 4B–D). Induced apoptosis Knockdown of PUMA in H9c2 cells exposed to H_2_O_2_ significantly increased BCL2 expression and decreased apoptosis (Figures 4A–E). In addition, miR-221/222 mimics significantly reduced ETS-1 expression in H_2_O_2_-treated cardiomyocytes (Figure 4F). Moreover, transfection with miR-221/222 mimic also significantly decreased H_2_O_2_-induced hypertrophy via ANP expression and flow cytometry (Figures 4F,G). Knockdown of ETS-1 in H9c2 cells exposed to H_2_O_2_ reduced ANP expression and cell hypertrophy (Figures 4G,H). Overexpression of PUMA was confirmed by immunofluorescent staining, and transfection of miR-221/222 mimics reduced PUMA expression (Figure 4I). TUNEL analysis indicated that transfection of miR-221/222 mimics reduced PUMA overexpression-induced apoptosis (Figures 4I,J). Overexpression of ETS-1 was also confirmed by immunofluorescent staining, which indicated that transfection of miR-221/222 mimics reduced ETS-1 expression (Figure 4K). Transfection with the miR-221/222 mimic decreased ETS-1 overexpression-induced hypertrophy compared to that in the control group (Figures 4K,L). Therefore, these results indicate that miR-221/222 regulates PUMA and ETS-1 expression, which is involved in apoptosis and hypertrophy.

### ADSC-Exo Reduced Apoptosis and Hypertrophy in H_2_O_2_-Treated H9c2 Cells via the AKT/NFκB Pathway

**FIGURE 5:**
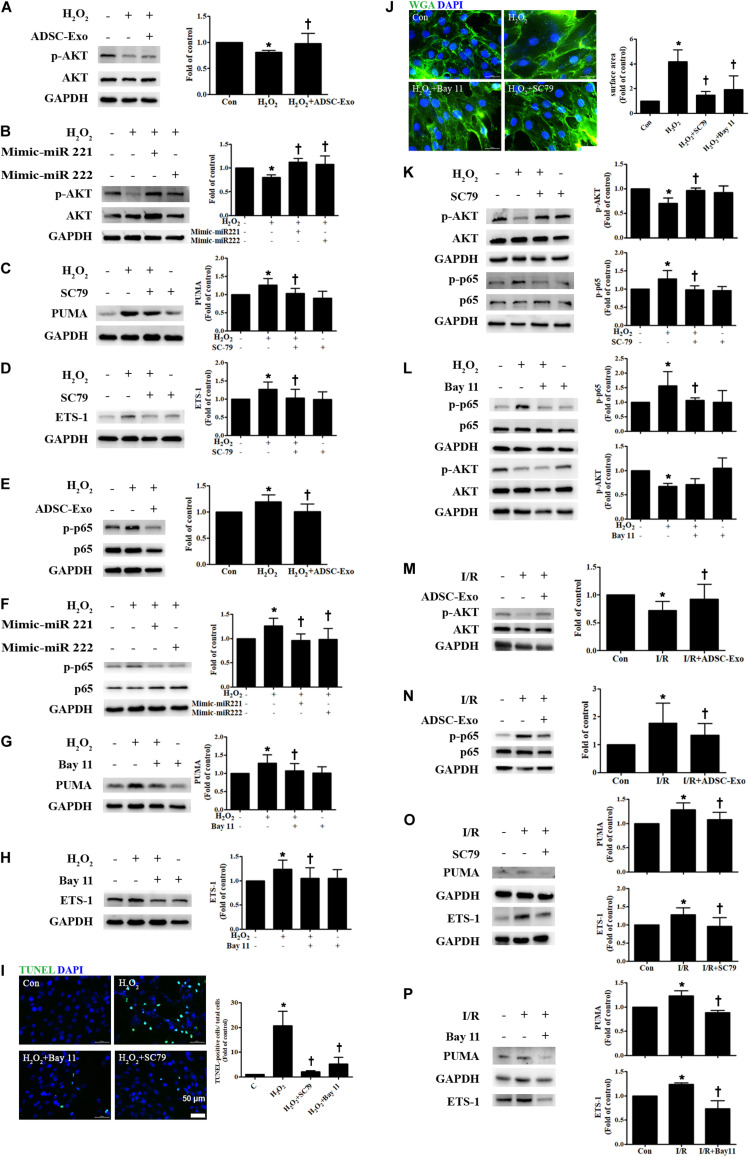
ADSC-Exo reduced apoptosis and hypertrophy in H_2_O_2_-treated H9c2 cells through the AKT/NFκB pathway. Cardiomyocytes were pretreated with or without ADSC-Exos (2 μg/mL) for 4 h and then exposed to 50 μM H_2_O_2_ for 48 h. **(A)** The effect on AKT was examined in H_2_O_2_-treated H9c2 cells. H_2_O_2_ reduced the phosphorylation of AKT when compared to that in control cells, while ADSC-Exo increased AKT expression, as indicated by Western blot analysis. **(B)** Transfection of miR-221/222 mimics significantly ameliorated H_2_O_2_-reduced AKT phosphorylation. **(C,D)** Pretreatment with SC79 (10 μM) for 1 h reduced H_2_O_2_-induced PUMA and ETS-1 expression in H9c2 cells. **(E)** The expression of NFκB in H_2_O_2_-treated H9c2 cells was examined by Western blot analysis. H_2_O_2_ increased the phosphorylation of NFκB p65 compared to that in control cells, while ADSC-Exo reduced the expression of p-p65. **(F)** Transfection of the miR-221/222 mimics significantly reduced the increased phosphorylation of p65 by H_2_O_2_. **(G,H)** Pretreatment with an NFκB inhibitor (Bay 11, 2.5 μM) for 1 h reduced H_2_O_2_-induced PUMA and ETS-1 expression in H9c2 cells. **(I,J)** Pretreatment with SC79 or with Bay 11 for 1 h decreased apoptosis and hypertrophy in H_2_O_2_-treated cardiomyocytes, as detected by TUNEL assay and WGA staining, respectively. Quantitative analysis of TUNEL-positive cells or cell surface area is shown. **(K,L)** SC79 reduced the phosphorylation of NFκB, while Bay 11 did not affect AKT phosphorylation. **(M,N)** I/R treatment significantly decreased p-AKT and increased p-p65 compared to that in control mice, while ADSC-Exo reversed these effects *in vivo*. **(O,P)** Mice treated with SC79 (10 mg/kg) or with Bay 11 (80 mg/kg) exhibited reduced expression of PUMA and ETS-1 in I/R-treated mice. Data are expressed as the mean ± SD (*n* = 3). *P < 0.05 vs. control; ^†^*P* < 0.05 vs. H_2_O_2_.

There is increasing evidence that AKT/NFκB plays a critical role in apoptosis and hypertrophy (Zhao et al., 2015; Luo et al., 2019). Cardiomyocytes were cultured alone or treated with H_2_O_2_. Compared to control cells, H_2_O_2_-treated H9c2 cells reduced levels of phosphorylated AKT, while ADSC-Exo-treated cells increased p-AKT expression (Figure 5A). Transfection of miR-221/222 mimics also significantly increased H_2_O_2_-reduced AKT phosphorylation (Figure 5B). To elucidate whether AKT pathway activation is necessary for the effects of ADSC-Exo on the apoptosis and hypertrophy of H_2_O_2_-treated H9c2 cells, the AKT activator SC79 was used during H_2_O_2_ treatment. SC79 reduced H_2_O_2_-induced PUMA and ETS-1 expression in H9c2 cells (Figures 5C,D). In addition, the expression of NFκB in H_2_O_2_-treated H9c2 cells was also examined. As shown in Figure 5E, H_2_O_2_ increased the phosphorylation of NFκB p65 compared to that in the control cells, whereas ADSC-Exo reduced p-p65 expression. Transfection of the miR-221/222 mimics also significantly reduced the increase in the phosphorylation of p65 by H_2_O_2_ (Figure 5F). To confirm the role of NFκB in ADSC-Exo-induced antiapoptotic and antihypertrophic effects in H9c2 cells, the cells were treated with H_2_O_2_ with or without the NFκB inhibitor Bay 11-7082 (Bay 11). As shown in Figures 5G,H, NFκB inhibitor reduced the expression of PUMA and ETS-1 in H_2_O_2_-treated H9c2 cells. TUNEL assay and WGA staining evaluated by fluorescence microscopy showed that treatment with an AKT activator or NFκB inhibitor also reduced the apoptosis and hypertrophy of H_2_O_2_-treated cardiomyocytes (Figures 5I,J). SC79 reduced the phosphorylation of NFκB, while Bay 11 did not affect the phosphorylation of AKT (Figures 5K,L). Furthermore, compared to the control treatment, I/R treatment significantly decreased p-AKT and increased p-p65 compared, whereas ADSC-Exo reversed these effects *in vivo* (Figures 5M,N). Treatment with an AKT activator or NFκB inhibitor also reduced the expression of PUMA and ETS-1 in I/R-treated mice (Figures 5O,P). Taken together, these findings indicate that ADSC-Exo with miR-221/222 prevent cardiac I/R injury through the AKT/NFκB pathway.

### ADSC-Exo Protected Against Myocardial I/R Injury in miR-221/222 KO Mice

**FIGURE 6:**
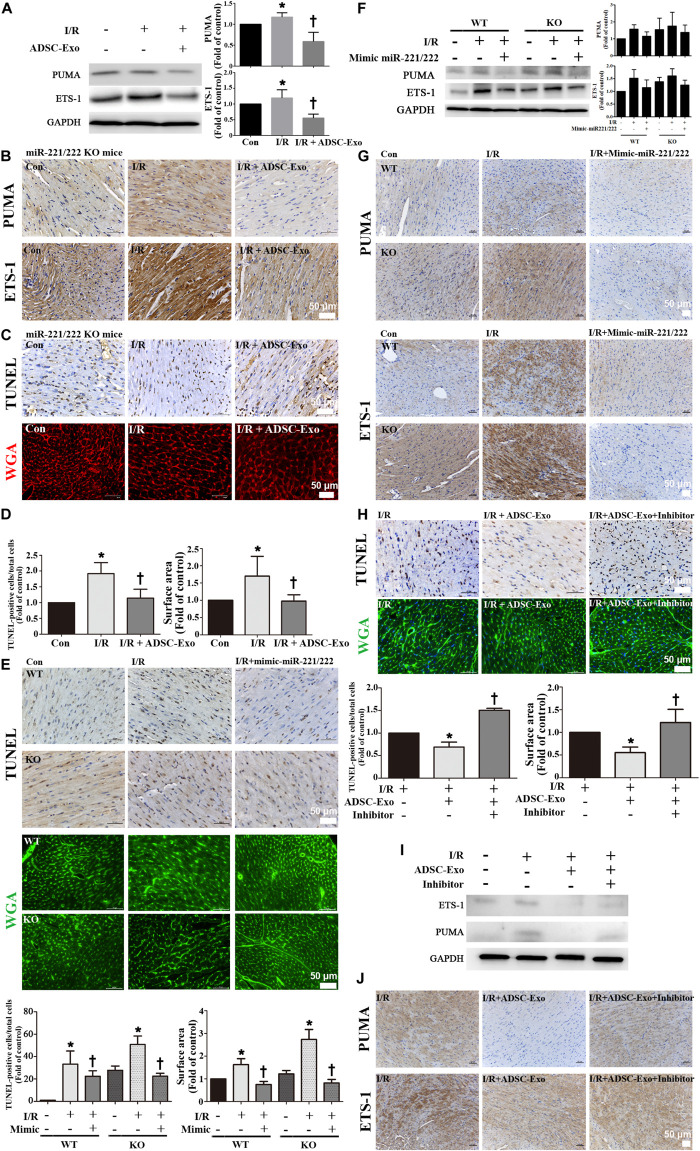
ADSC-Exo has protective effects on myocardial I/R injury in miR-221/222 KO mice. miR-221/222 KO mice were subjected to ischemia for 30 min followed by reperfusion for 3 h. In the I/R + ADSC-Exo-treated miR-221/222 KO mouse model, 25 min after occlusion, ADSC-Exo (100 μg protein in 50 μL), miR-221/222 mimics or inhibitors (50 pmole in 50 μL) were injected uniformly intramuscularly into the border zone of the anterior wall of the left ventricle at five positions, followed by reperfusion for 3 h. **(A)** The levels of PUMA and ETS-1 protein were evaluated by Western blot analysis. **(B)** The effects of ADSC-Exo on PUMA and ETS-1 expression in miR-221/222 KO mice. **(C,D)** The effects of ADSC-Exo on apoptosis and hypertrophy were examined by TUNEL assay and WGA staining, respectively, in miR-221/222 KO mice. The results from the quantitative analysis of TUNEL-positive cells or cell surface area are shown. **(E)** The effects of the transfection of miR-221/222 mimics on apoptosis and hypertrophy were examined by TUNEL assay and WGA staining, respectively, in WT and miR-221/222 KO mice. Quantitative analysis of TUNEL-positive cells or cell surface area is shown. **(F,G)** The effects of the transfection of miR-221/222 mimics on the expression of PUMA and ETS-1 were examined by Western blot analysis and by immunohistochemistry in WT and miRNA-221/222 KO mice. **(H)** The effects of the transfection of miR-221/222 inhibitors on apoptosis and hypertrophy were examined by TUNEL assay and WGA staining, respectively, in WT mice. The results of the quantitative analysis of TUNEL-positive cells and cell surface area are shown. **(I,J)** The effects of the transfection of miR-221/222 inhibitors on the expression of PUMA and ETS-1 were examined by Western blot and by immunohistochemistry in WT mice. Data are expressed as the mean ± SD (*n* = 3). Brown color: apoptotic cells; blue color: nuclei. Red or green color: cell boundary. *P < 0.05 vs. the control group; ^†^*P* < 0.05 vs. I/R group. Scale bar, 50 μm.

There was a significant increase in PUMA and ETS-1 expression after I/R treatment in miR-221/222 KO mice, and ADSC-Exo alleviated the I/R-induced upregulation of PUMA and ETS-1 (Figures 6A,B). In addition, ADSC-Exo treatment reduced apoptosis and hypertrophy in cardiomyocytes, as indicated by TUNEL assay and WGA staining, respectively, in I/R-treated miR-221/222 KO mice (Figures 6B–D). Consistent with the results of ADSC-Exo treatment, miR-221/222 transfection significantly reduced apoptosis according to TUNEL assay and hypertrophy according to WGA staining compared to that in the I/R group (Figure 6E). In addition, the PUMA and ETS-1 levels of the I/R group were upregulated, while those of the I/R+mimic-miR-221/222 group were restored (Figure 6F). Similarly, this result was confirmed by immunohistochemistry (Figure 6G). In contrast, the I/R+ADSC-Exo+miR-221/222 inhibitors group did not have reduced apoptosis and hypertrophy compared with that in the I/R group (Figure 6H). The expression of PUMA and ETS-1 was upregulated in the I/R group and was not affected by the I/R+ADSC-Exo+ miR-221/222 inhibitors group (Figure 6I). The results were confirmed by immunohistochemistry (Figure 6J). These findings strongly support that ADSC-Exo can prevent myocardial I/R injury through miR-221/222 *in vivo*.

## Discussion

Apoptosis and hypertrophy of cardiomyocytes are important components of I/R-induced heart injury. In this study, we found that myocardial apoptosis and hypertrophy-related protein expression increased after I/R treatment. However, treatment with exosomes from ADSCs reversed these effects. We have also demonstrated that cardioprotective effects are achieved through high amounts of miR-221/222 targeting proteins PUMA and ETS-1.

The loss of cardiomyocytes during myocardial I/R is mainly due to cell death and ischemic stress in surviving myocytes, which triggers left ventricle remodeling and leads to hypertrophy (Konstantinidis et al., 2012). Cell death is primarily caused by apoptosis and necrosis. In addition, cardiac hypertrophy is characterized by an increase in the size of individual cardiomyocytes. Although hypertrophy was initially considered a compensatory response to balance wall pressure during systole, there is increasing experimental evidence that hypertrophy activates maladaptive cellular processes and promotes disease progression (Bisping et al., 2014). Indeed, cardiac hypertrophy is a powerful and independent risk factor for poor prognosis of heart disease (Hawkins et al., 2007). Since cardiomyocytes are terminally differentiated and have little potential for division, reducing the apoptosis and hypertrophy of cardiomyocytes after injury has potential therapeutic value (Brown and Griendling, 2015). Previous studies have demonstrated that PUMA and ETS-1 contribute to the apoptosis and hypertrophy of cardiomyocytes, respectively (Zhou et al., 2016; Li Y. et al., 2017). Several researchers have proposed various pathways of cardiomyocyte apoptosis by activating PUMA, a p53-responsive BH3-only protein (Yang et al., 2014, 2019). Lack of PUMA reduced the infarct size of isolated perfused hearts receiving I/R (Toth et al., 2006). PUMA ablation attenuated cardiac dysfunction in a mouse heart failure model by reducing apoptosis (Mandl et al., 2011). Suppression of PUMA can inhibit apoptosis *in vivo* and protect against neonatal hypoxic-ischemic brain injury. In addition, the pathologic process of cardiomyocyte hypertrophy is highly related to the expression of ETS-1. Ang II infusion induced an increase in heart size and ventricular wall thickness in mice, but these effects were significantly diminished in global ETS-1 knockout mice (Zhan et al., 2005; Xu et al., 2019). Our current research shows that adding H_2_O_2_ to cultured cardiomyocytes can increase the levels of PUMA and ETS-1 and that inhibiting PUMA and ETS-1 can reduce apoptosis and hypertrophy, respectively. We also show that I/R increases myocardial apoptosis and hypertrophy by TUNEL and WGA staining, respectively. Moreover, I/R treatment significantly reduces BCL2 levels and increases the expression of p-p53, caspase-3, PUMA (apoptotic markers), ANP, and ETS-1 (hypertrophic markers), while ADSC-Exo treatment significantly reverses this effect. ADSC-Exo reduces apoptosis and hypertrophy and can be beneficial for I/R-induced damage.

Mesenchymal stem cells are more suitable for the treatment of ischemic diseases because they involve fewer ethical issues, higher self-renewal ability, and lower immunogenicity. Our studies have previously reported the potential efficacy of human umbilical cord mesenchymal stem cells in repairing vascular injury (Wang et al., 2009; Shen et al., 2013; Liang et al., 2015). Furthermore, ADSCs are attractive because they are easy to obtain, rapidly expand *in vitro*, and can be collected from large amounts of adipose tissue. Importantly, a recent study showed that the observed therapeutic effects are primarily mediated by stem cell secretion (Harrell et al., 2019). Exosomes are the most effective active paracrine components, play an important role in intercellular communication, and have great potential for repairing damaged tissue (Lai et al., 2010). Exosomes carry complex miRNAs and proteins and may affect many cellular processes and pathways (Théry et al., 2009). Past studies have shown that exosomes collected from MSCs play a key role in protecting against heart and kidney damage caused by I/R (Lin et al., 2016; Zhang et al., 2016). In addition, exosomes are potentially safer because their application is feasible and will reduce manufacturing and storage costs. ADSC-Exo plays a beneficial role by increasing the migration of human umbilical vein endothelial cells and the formation of capillary networks *in vitro* (Han et al., 2018). Our previous study demonstrated that ADSC-Exo treatment increased flap survival and capillary density compared to that with I/R through the IL-6 pathway (Pu et al., 2017). Another study also showed that ADSC-Exo protected the myocardium from I/R injury via the Wnt/β-catenin pathway (Cui et al., 2017). We found here that ADSC-Exo can be absorbed and internalized by cardiomyocytes to inhibit apoptosis and hypertrophy by reducing the expression of PUMA and ETS-1 in H_2_O_2_-treated cultured cardiomyocytes and I/R-treated mice. Based on these findings, ADSC-Exo can be used to therapeutically regulate cardiomyocyte function.

Dysregulation of miRNA expression has been reported in numerous studies involving various pathophysiological processes, including I/R and heart failure (Barraclough et al., 2019). In these studies, miR-221/222 has previously been reported to play an essential role in inflammation, cardiovascular function and tissue metabolism (Chistiakov et al., 2015; Song et al., 2017; Verjans et al., 2018). miR-221/222 is very abundant in ADSC-Exo and will be selected for further research. miR-221 and miR-222 are highly homologous miRNAs encoded on the X chromosome and form miR-221-222 clusters (Song et al., 2017). Compared with that in healthy controls, miR-221-3p and miR-222-3p expression was significantly reduced in atherosclerotic patients, suggesting that downregulation of these two miRNAs may be related to the atherosclerotic process (Zhang et al., 2014). Apo E regulates p27 expression through miR-221/222, which limits the proliferation of smooth muscle cells (Kothapalli et al., 2013). ICAM-1 mRNA expression increases while miR-221 expression decreases in the aorta and heart (Duan et al., 2013). Systemic inhibition of miR-221/222 *in vivo* leads to severe heart damage, increased cardiac viral load, and prolonged viremia during viral myocarditis (Corsten et al., 2015). Using bioinformatics predictions, miR-221/222 complementary binding sites have been identified in the 3′ UTRs of PUMA mRNA and ETS-1 mRNA, which have antiapoptotic and antihypertrophic properties, respectively. Overexpression of miR-221/222 in glioblastoma induces cell survival, whereas knockdown of these miRs leads to increased apoptosis by upregulating PUMA (Zhang et al., 2010). In this study, we found that I/R or H_2_O_2_ conditions reduced miR-221/222 expression levels. Treatment with exosomes containing a large amount of miR-221/222 or transfection of miR-221/222 significantly reduced PUMA and ETS-1 expression, suggesting that ADSC-Exo can reduce I/R-induced myocardial damage by downregulating PUMA and ETS-1. We demonstrated that miR-221/222 can act as an upstream regulator of PUMA and ETS-1, thereby regulating cellular apoptotic and hypertrophic processes.

The pathogenesis of heart injury involves multiple intracellular signaling cascades, including the ROS, AKT, and transcription factor (NF-κB) pathways (Ma et al., 2014). Considering the involvement of ADSC-Exo in the reduction of I/R-induced apoptosis and hypertrophy, it is of great significance to regulate these signals. The AKT signaling pathway plays an essential role against I/R injury by regulating proapoptotic signals, inflammation and ROS (Li Q. et al., 2019). Oxidative stress-induced cell apoptosis is due to AKT dysfunction and NFκB upregulation (Wang et al., 2019). JNK/NFκB is involved in myocardial I/R-induced apoptosis (Zhang et al., 2019). TNF-α activates NF-κB through ROS generation and the AKT pathway to prevent cardiomyocyte death (Tang et al., 2018). The NFκB inhibitor Bay 11-7082 has been shown to inhibit left ventricular remodeling after MI (Wang et al., 2017). Another study confirmed that AKT phosphorylation was significantly reduced in the hearts of high-fat diet-fed mice following I/R injury (Samidurai et al., 2019). The effect of AKT and p-p65 expression is considered a potential therapeutic target for cardiac I/R injury (Yuan et al., 2015). Consistent with some of these previous studies, p-AKT was decreased and p-p65 was increased in H_2_O_2_-treated H9c2 cells and I/R-treated hearts in the present study. We also demonstrated that the AKT activator and the NF-κB inhibitor significantly reduced PUMA expression and cell apoptosis. They also reduced ETS-1 expression and cell hypertrophy. Previous studies suggested that miRNA-regulated cytokine production is mediated by modulating AKT/NF-κB (Zhao et al., 2019). miRNA-29a regulates lipopolysaccharide-induced inflammatory responses in murine macrophages through the Akt1/NF-κB pathway (Tang et al., 2017). miR-221/222 promotes cancer stem-like cell properties and tumor growth of breast cancer by targeting PTEN and sustaining Akt/NF-κB/COX-2 activation (Li B. et al., 2017). Furthermore, the present study demonstrated that administration of exogenous ADSC-Exo and transfection of miR-221/222 mimics can activate AKT signaling and reduce NF-κB expression to provide protection. Thus, our findings indicate that ADSC-Exo with miR-221/222 play a critical role in the prevention of cardiac I/R-induced apoptosis and hypertrophy by targeting PUMA and ETS-1. This protective effect is mediated through the AKT/NFκB pathway.

Myocardial I/R damage leads to apoptosis and hypertrophy. We demonstrate that ADSC-secreted exosomes have a positive role in alleviating I/R injury. Our findings indicate that ADSC-Exo can reduce I/R-induced damage through the miR-221/miR-222/PUMA/ETS-1 axis. These data provide strong *in vitro* and *in vivo* evidence that ADSC-Exo has broad prospects for clinical application in I/R injury.

## Data Availability Statement

The raw data supporting the conclusions of this article will be made available by the authors, without undue reservation.

## Ethics Statement

The animal study was reviewed and approved by the National Taiwan University College of Medicine Instructional Animal Care and Use Committee (IACUC Approval No: 20150502).

## Author Contributions

T-CL, T-LL, Y-CCha, and Y-CChe: conception and design, collection and/or assembly of data, data analysis and interpretation, and final approval of the manuscript. S-RL, S-WL, C-MP, and J-ST: provision of study material and final approval of the manuscript. Y-LC: conception and design, financial support, data analysis and interpretation, manuscript writing, and final approval of the manuscript. All authors contributed to the article and approved the submitted version.

## Conflict of Interest

The authors declare that the research was conducted in the absence of any commercial or financial relationships that could be construed as a potential conflict of interest.
