# Supplementary material for: MicroRNA-221/222 Mediates ADSC-Exosome-Induced Cardioprotection Against Ischemia/Reperfusion by Targeting PUMA and ETS-1
Source: Front Cell Dev Biol. 2020 Dec 3;8:569150. doi: 10.3389/fcell.2020.569150 (PMC7744807; doi:10.3389/fcell.2020.569150)
Supplement: Supplementary file 1 [file Data_Sheet_1.PDF]

MicroRNA-221/222 mediates ADSC-Exosome-induced cardioprotection against ischemia/reperfusion by targeting PUMA and ETS-1

Tsai-Chun Lai, Tzu-Lin Lee, Yu-Chun Chang, Yu-Chen Chen, Shu-Rung Lin, Shu-Wha Lin, Chi-Ming Pu, Jaw-Shiun Tsai and Yuh-Lien Chen*

* Correspondence:

Yuh-Lien Chen

ylchenv@ntu.edu.tw

MATERIALS AND METHODS

Characterization of ADSC-Exo

The isolated ADSC-Exo and ADSCs were characterized using the exosome biomarkers CD63 and CD9 and mesenchymal stem cell maker CD73, respectively, by Western blot. The morphology of ADSC-Exo was observed under a transmission electron microscope (Hitachi, Tokyo, JP). Electron Microscopy was performed after a standard staining procedure with 0.5% uranyl acetate (Théry et al., 2006). Nanoparticle tracking analysis (NTA) is based on the principle that the rate of Brownian movement of nanoparticles in solution is related to their size (Dragovic et al., 2011). According to this method, a 488 nm laser light is directed at a fixed angle to the vesicle suspension, and the scattered light is captured using a microscope and high-sensitivity camera. By tracking the movement of individual nanoparticles over time, the software can calculate their diameter. Exosome observation was examined using a Nanosight NS300 (Malvern Panalytical Ltd, Malvern, UK) with constant flow injection (Dragovic et al., 2011).

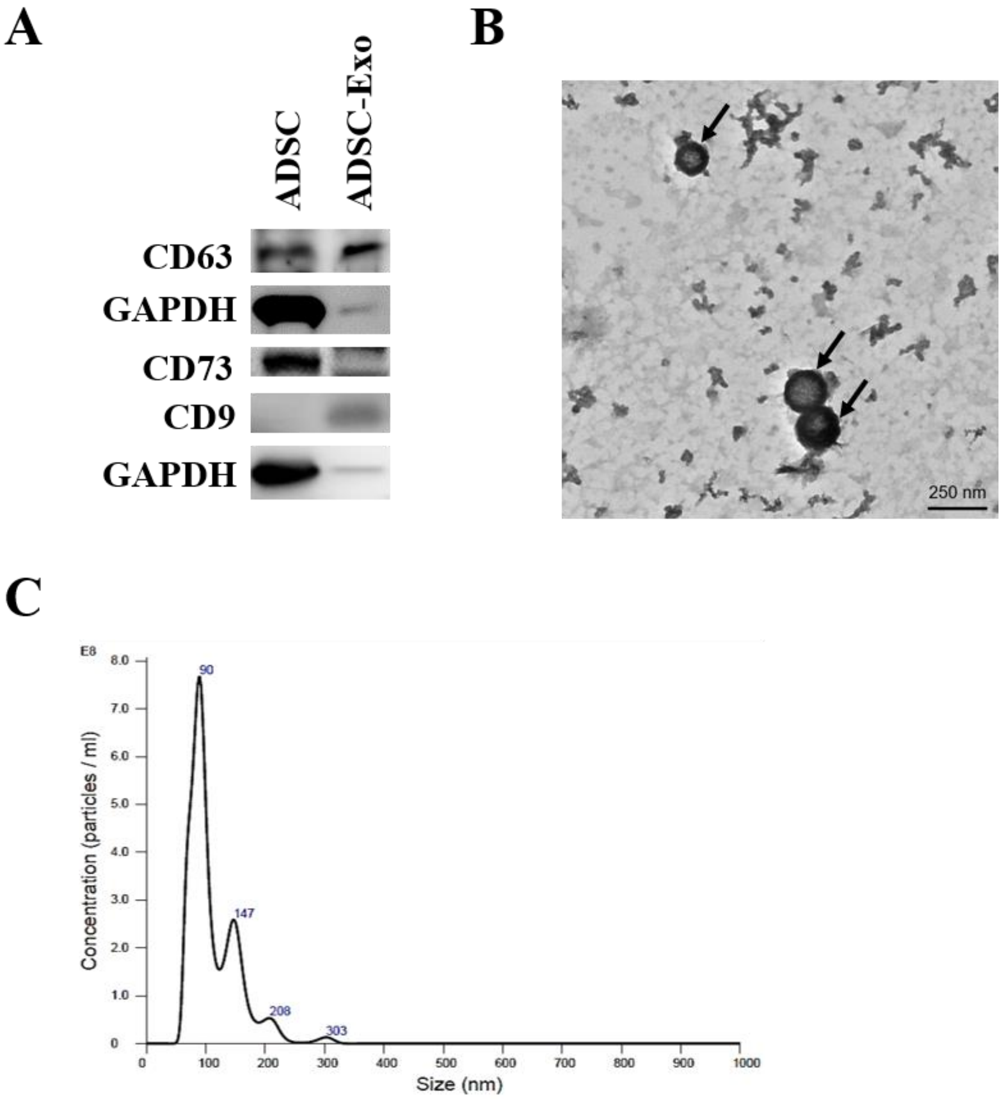

FIGURE S1 Characterization of ADSC-Exo. **(A)** Western blot analysis was used to evaluate the expression of mesenchymal stem cell marker CD73, and exosome biomarker CD63 and CD9 in ADSC cell lysate and ADSC-Exo, respectively. The level of CD63 and CD9 was strongly expressed in ADSC-Exo, while CD 73 was strongly expressed in ADSC cell lysate. **(B)** The morphology and size of ADSC-Exo (arrows) were observed by TEM. The morphology of ADSC-Exo showed central depression with “cup-shape”. Scale bar, 250 nm. **(C)** ADSC-Exo were subjected to NTA analysis. The average size of ADSC-Exo is equal to 112 nm, and the mode value (major peak) is equal to 90 nm.

REFERENCES

Dragovic, R.A., Gardiner, C., Brooks, A.S., Tannetta, D.S., Ferguson, D.J., Hole, P., et al. (2011). Sizing and phenotyping of cellular vesicles using nanoparticle tracking analysis. *Nanomedicine* 7**,** 780-788. doi: 10.1016/j.nano.2011.04.003

Théry, C., Amigorena, S., Raposo, G., and Clayton, A. (2006). Isolation and characterization of exosomes from cell culture supernatants and biological fluids. *Curr Protoc Cell Biol* Chapter 3**,** Unit 3.22. doi: 10.1002/0471143030.cb0322s30
